# Performance in the Six-Minute Walking Test Does Not Discriminate Excessive Erythrocytosis Patients in a Severe Hypoxic Environment

**DOI:** 10.3390/ijerph21091119

**Published:** 2024-08-25

**Authors:** Rossela Alejandra Rojas-Chambilla, Kely Melina Vilca-Coaquira, Jeancarlo Tejada-Flores, Henry Oscar Tintaya-Ramos, Mariela Mercedes Quispe-Trujillo, Ángel Gabriel Calisaya-Huacasi, Solanyela Anny Quispe-Humpiri, Yony Martin Pino-Vanegas, Alberto Alcibiades Salazar-Granara, Ana Lucía Tácuna-Calderón, Nancy Mónica García-Bedoya, Moua Yang, Ginés Viscor, Iván Hancco-Zirena

**Affiliations:** 1 Facultad de Medicina Humana, Universidad Nacional del Altiplano, Puno 21000, Peru; rosselarojas@gmail.com (R.A.R.-C.); kevilcac@est.unap.edu.pe (K.M.V.-C.); jeancarlotejada@gmail.com (J.T.-F.); hedsoytintaya@gmail.com (H.O.T.-R.); marielaquispet94@gmail.com (M.M.Q.-T.); angelcalisayahuacasi@gmail.com (Á.G.C.-H.); soquispeh@est.unap.edu.pe (S.A.Q.-H.); 2 Asociación Científica de Estudiantes de Medicina (ACEM), Puno 21000, Peru; 3 Escuela Profesional de Educación Física, Facultad de Ciencias de la Educación, Universidad Nacional del Altiplano, Puno 21000, Peru; ympino@unap.edu.pe; 4 Centro de Investigación en Medicina de Altura (CIMA), Facultad de Medicina Humana, Universidad de San Martín de Porres, Lima 15024, Peru; asalazarg@usmp.pe (A.A.S.-G.); atacunac@usmp.pe (A.L.T.-C.); ngarcia@unap.edu.pe (N.M.G.-B.); 5 Division of Hemostasis and Thrombosis, Beth Israel Deaconess Medical Center, Harvard Medical School, Boston, MA 02215, USA; 6 Bloodworks Northwest Research Institute, Seattle, WA 98102, USA; 7 Division of Hematology and Oncology, Department of Medicine, School of Medicine, University of Washington, Seattle, WA 98102, USA; 8 Secció de Fisiologia, Departament de Biologia Cel·lular, Fisiologia i Immunologia, Facultat de Biologia, Universitat de Barcelona, E-08028 Barcelona, Spain

**Keywords:** excessive erythrocytosis, high-altitude native, La Rinconada, Andes, six-minute walking test, physical performance, hypobaric hypoxia, chronic mountain sickness, hemoglobin, hematocrit

## Abstract

Background: Chronic exposure to severe hypoxia causes an increase in hematocrit (Hct) and hemoglobin concentration ([Hb]), which can lead to excessive erythrocytosis (EE) and impact physical performance. This work aims to determine the differences in the six-minute walking test (6MWT) between EE and healthy subjects residing at more than 5000 m. Methods: A prospective, cross-sectional study was performed on 71 men (36 healthy and 25 suffering from EE) living in La Rinconada, Peru (5100 m). Basal levels of [Hb] and Hct were obtained. All the subjects performed the 6MWT, and distance reached, vital signs, dyspnea, and fatigue (Borg scale) at the end of the test were recorded. Results: The average [Hb] and Hct levels in the control group were 18.7 ± 1.2 g/dL and 60.4 ± 7.1%, respectively, contrasting with EE subjects, who showed 23.4 ± 1.6 g/dL and 73.6 ± 5.9% (*p* < 0.001). However, no statistically significant differences were observed in BMI or other anthropometric parameters. At the end of the 6MWT, the distance traveled and vital constants were similar between both groups, except for arterial oxygen saturation, which was consistently lower in subjects with EE throughout the test. Conclusion: EE does not significantly affect 6MWT performance at high altitudes, nor the hemodynamic control during moderate aerobic exercise of subjects who live permanently in a severely hypoxic environment.

## 1. Introduction

A hypoxic environment produces a series of physiological changes in the inhabitants of high-altitude regions due to the decreased oxygen availability. In the Andean region, one of the most conspicuous changes is an increase in red blood cell count, accompanied by elevated levels of hemoglobin concentration ([Hb]) and hematocrit (Hct), and subsequent arterial pulmonary hypertension (PHT) [1], thus preventing those inhabitants from carrying out intense physical activity [2,3]. These physiological changes that occur from 2500 m are especially prominent in permanent residents of areas located at more than 5000 m above sea level, because the barometric pressure is much lower in relation to sea level. The barometric pressure change will produce a situation of severe hypoxia, which will have a significant impact on tissue oxygenation [4]. It is also known that during physical activity the demand for oxygen increases depending on the intensity of the physical exercise and this could have a negative impact on daily activities [3].

The hypoxic environment is associated with a series of changes at all levels, including the cardiovascular, respiratory, and metabolic systems. An increase in sympathetic nervous activity has also been described, which produces a reduction in exercise performance. These phenomena occur acutely with exposure to hypoxia [5]. Changes also occur at different levels during chronic exposure; however, the adaptation process allows for better tolerance for the lack of oxygen due to a series of compensatory mechanisms [6]. Despite these changes there are reference values that have not been established for health and physical examinations at high altitude, including the six-minute walking test (6MWT). A knowledge gap exists regarding whether the parameters used for the 6MWT are similar or different in high altitude residents compared to residents at sea level. The adaptation process has helped residents achieve better tolerance to physical effort at a high altitude; however, it is unclear how high-altitude residents tolerate submaximal exercise under severe hypoxic conditions.

To objectively estimate the ability to exercise, several tests are available ranging from stair climbing, performing the cardiopulmonary exercise test, and the 6MWT [7]. A basic form of locomotion is walking, which requires the coordination of numerous muscles that allow for forward movement without losing body balance. At the same time, this motor function requires energy expenditure that will depend on the speed at which one walks [8]. The distance and speed that a healthy person can walk is determined by several factors, such as age, sex, height, motivation, and lifestyle, among others [9]. Physical exertion would be required for occupational and daily living activities, as well as for rapid emergency situations. Insufficient oxygen availability could hamper these activities. The presence of a health problem presents factors that limit the speed with which one can walk and becomes even more complex when there is deterioration of any system involved in locomotion. This can limit the general ability to walk a specific distance at a specific time [9]. Conditions of the environment, such as hypoxia, can also influence walking, limiting the ability to satisfy increased oxygen needs during the exertion of physical effort.

Many physiologic and performance evaluations that rely on the environmental oxygen level, the respiratory system, and tissue oxygenation do not yield normal values, depending on the altitude of residence. This is especially true in very high-elevation locations such as La Rinconada, which is currently considered the highest inhabited city in the world (5100 m). The 6MWT is one such example that measures physiological output. The 6MWT evaluates, through distance walked, the functional capacity to perform submaximal exercise and the coordination of body systems during exercise [7,10]. This method can easily estimate functional capacity at low cost and is performed at the subject’s own pace. It does not require specific equipment or prior training [10,11].

When applying the 6MWT to those high-altitude living patients, it is unknown whether disproportionate increases in [Hb] and Hct values could affect the results. Despite their prolonged stay under these conditions, several physiologic adjustments allow inhabitants to accommodate to the high-altitude environment. High [Hb] and Hct values typical of high-altitude acclimatization could alter the results and lead to misinterpretation due to the increased blood viscosity and potential decrease in tissue perfusion during physical activity. The increase in blood viscosity and decrease in tissue perfusion could mechanistically be explained by the elevated red blood cell mass hindering blood flow and limiting oxygen delivery [11,12].

The increase in the number of red blood cells stimulated by hypoxia helps to improve oxygen transport to tissues [6]; however, when it exceeds certain values it could have negative effects on tissue oxygenation altering blood circulation [11,12,13,14,15,16,17]. This is an issue with the high values presenting in excessive erythrocytosis (EE). When [Hb] levels are above 21 g/dL in men and 19 g/dL in women, this sign is accompanied by other respiratory, neurological or cardiovascular symptoms constituting a pathologic entity known as chronic mountain sickness (CMS) [18]. We hypothesize that these hematological changes could interfere with the execution of the 6MWT, altering the normal range parameters and leading to inappropriate interpretation. Furthermore, this could have a significant impact on daily activities and the development of submaximal physical activity in the individuals.

The main objective of this study is to determine the differences in 6MWT performance and the hemodynamic response during this test on subjects who permanently reside at more than 5000 m of altitude with and without EE. The 6MWT is a simple, low-cost, sensitive test, mainly used in the evaluation of and follow-ups regarding the therapeutic response of patients with respiratory and cardiovascular diseases [17,19]. We applied this test to evaluate apparently healthy patients with and without EE to determine whether the high increase in [Hb] and Hct interfere with the 6MWT performance in a severe hypoxic environment.

## 2. Materials and Methods

### 2.1. Participants

The study was carried out in La Rinconada, located at more than 5100 m above sea level in the Puno Region within southern Peru, considered the highest city in the world with more than 60,000 inhabitants [20]. Seventy-one people, all male, apparently healthy and meeting the inclusion criteria were evaluated. All subjects who voluntarily agreed to participate in the study were considered. Because of this, and because the variance was not previously known, power analysis could not be performed to calculate the sample size. Only a representative group was divided according to the [Hb] and Hct values with EE and without EE. Information on age, sex, weight, height, length of residence in La Rinconada, and place of origin was obtained during a collective medical consultation organized by the local Miners’ Association.

For blood sampling, aseptic measures were taken by using an alcohol-moistened cotton swab on the tip of the patient’s middle or ring fingers, followed by capillary puncture using a sterile lancet. After removing the lancet, a waiting period allowed for the spontaneous formation of a blood drop. The initial two drops were removed with a cotton swab to prevent errors, ensuring the third drop was of sufficient volume to fill a micro cuvette for [Hb] measurement and a capillary tube for Hct determination. The microcuvette with the blood sample was positioned for analysis using a HEMOCUE HB 201+ portable hemoglobinometer, employing the azide methemoglobin method within a measurement range of 0 to 25.6 g/dL. Hematocrit measurements were conducted using a Hemata Stat II microcentrifuge from EKF Diagnostics, involving the placement of the tube for centrifugation and subsequent measurements. The current international consensus on chronic mountain sickness [18] was used to consider a case of excessive erythrocytosis (EE).

### 2.2. Six-Minute Walking Test

The 6MWT was performed according to the protocol established by the American Thoracic Society [7]. The individuals responsible for implementing the test were trained to avoid errors during the examination. The test was carried out in a large environment consisting of a 60-meter-long course without any obstacles or conditions that could alter the examination. Vital sign measurements included the following: heart rate (HR), oxygen saturation (SpO_2_), systolic blood pressure (SBP), and diastolic blood pressure (DBP). For the initial measurements, the subjects rested for 10 min in the sitting position. After, the subjects immediately performed the 6MWT. Recordings were made just at the end of the test and after 3 and 5 min of recovery. SpO_2_ and HR were measured every minute using a pulse oximeter. The pulse oximeter was placed so the values could be observed every minute without interfering with the 6MWT.

The Borg scale was used to evaluate the perception of dyspnea and fatigue. A pulse oximeter was placed on the index finger. A Nellcor^®^ Oximax^®^ N-65 brand pulse oximeter (Digicare Biomedical Technology, Inc., Boynton Beach, FL 33426, USA) with a resolution of 1% saturation and a heart rate range of 30 to 235 beats per minute was used. A Riester brand Ri-Champion adult digital blood pressure monitor was used to measure blood pressure with an arm sphygmomanometer able to measure from 30 to 280 mm and a heart rate from 40 to 200 beats per minute. A Camry model EB9068-59 digital scale and a measuring rod were used to estimate the weight and height of the subjects evaluated and subsequently calculate the body mass index (BMI). The measuring instruments had previously been calibrated and the respective verification was made by comparing the measurements with similar instruments [17]. At the end of the test, the distance traveled in 6 min was recorded.

### 2.3. Statistical Analysis

Descriptive data are reported as mean ± standard deviation for continuous variables. Student’s *t*-test was used to determine the relationship between two variables. An α level of 0.05 was established for testing null hypotheses. Data analysis was performed using the IBM SPSS v26 statistical package.

## 3. Results

Seventy-six young, apparently healthy subjects agreed to participate in this study, but, finally, a total of 71 subjects completed the protocol. All of the subjects were male: 36 had no EE, while 35 presented EE. The subjects without EE had a BMI similar to subjects with EE. The average [Hb] and Hct levels in the control group were lower than in subjects with EE (*p* < 0.001). No significant differences were observed in the other variables studied, including body weight and height (Table 1).

Blood pressure and heart rate in all of the subjects were also evaluated at rest and before testing their sub-maximal physical capacity. Resting SBP values were higher in patients with EE by approximately 10 mmHg (Table 2) Statistical significance was marginal between individuals with EE and the control groups (*p* = 0.052). Likewise, DBP values remained within normal limits with no statistical difference in both groups (*p* = 0.395). The baseline vital signs showed similarities in some variables measured between subjects with and without EE (Table 2). The SpO_2_ variance was also similar in both groups; however, subjects with EE maintained lower final SpO_2_ levels as compared to control subjects (*p* = 0.001).

**Figure 1:**
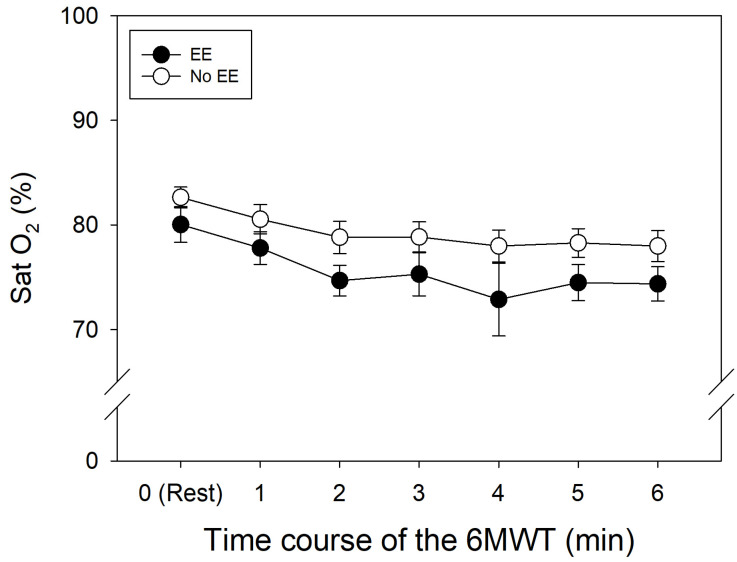
Oxygen saturation over time along 6MWT in patients with and without EE.

The 6MWT was performed after measurements of baseline parameters. During the 6MWT, SpO_2_ levels in both the control group and EE group showed similar decreases over time relative to resting conditions (Table 3, Figure 1). As expected, HR increased in both of the groups during the test, with a final HR of roughly 119 bpm.

The distance traveled as well as dyspnea and fatigue during the 6MWT did not offer important differences. The total distance traveled was slightly greater in subjects without EE compared to subjects with EE (Table 4), which represents only a 1.58% improvement in the control group relative to the subjects with EE. The difference did not reach statistical significance (*p* = 0.433).

## 4. Discussion

La Rinconada is a city located above 5100 m with many of the residents working in the existing gold mines in the area. The oxygen pressure at La Rinconada can be as low as 50% relative to sea level and contributes significantly to the level of EE in the residents [21]. Here, the prevalence of EE is 44.1% and 13.9% for CMS [22]. The residents with EE are chiefly characterized by the increase in [Hb] and Hct levels. The severe environmental conditions of this place could negatively influence work performance, since in many cases mining demands a high level of physical effort [22,23].

The 6MWT is very frequently used for the evaluation and monitoring of patients with cardiac and respiratory diseases, as well as the evaluation of their response to treatment [23]. The 6MWT reference values were developed for a low altitude normoxic environment. Despite its usefulness, there is no information regarding the values in severe hypoxic environments, such as in the case of La Rinconada. In the present work, we have carried out this procedure to determine possible changes in performance in conditions of severe hypoxia and to determine whether the excessive increase in [Hb] and Hct, as in the case of EE, could alter the results of this test. No studies have been carried out to determine whether the excessive increase in Hb and Hct can alter tests to measure physical output, especially the tests that depend on oxygen transport. In addition, many of the auxiliary tests and complementary methods have not been studied, nor have they been established for individuals living at high altitude.

The findings in the present study indicate that in subjects with and without EE, the performance in the 6MWT was similar, and that the high-altitude environment may not have a major role in altering the response to the 6MWT. These findings are in line with previous reports that hypobaric hypoxia does not alter the hemodynamic response during the exertion of physical effort [23,24] and is probably due to the acclimatization process in the subjects, since they remain within the region for long periods of time. The results in this study indicate that DBP and SBP values were slightly increased in the subjects with EE relative to the control group, and that the 6MWT did not impact these vital signs. Adaptive responses to the high-altitude environment [25] may contribute to the minimal differences between the two groups. The modest differences may allow the residents to lead a normal daily life, potentially through physiologic changes inherent to the altitude adaptation process [6]. Mechanistically, this may be due to a reduction in peripheral resistance that allows blood perfusion to be maintained in the organs where a high level of oxygenation is required during physical activity [26]. The adaptation process included morphological and functional changes at, most importantly, the level of the heart, pulmonary circulation, gas exchange, erythrocyte elevation, and greater capillarity [27]. The increase in the number of erythrocytes also improves the transport of oxygen to peripheral tissues [28]. Subjects who present EE have mechanisms that promote normal blood flow despite the excessive increase in [Hb] and Hct levels [29].

**Figure 2:**
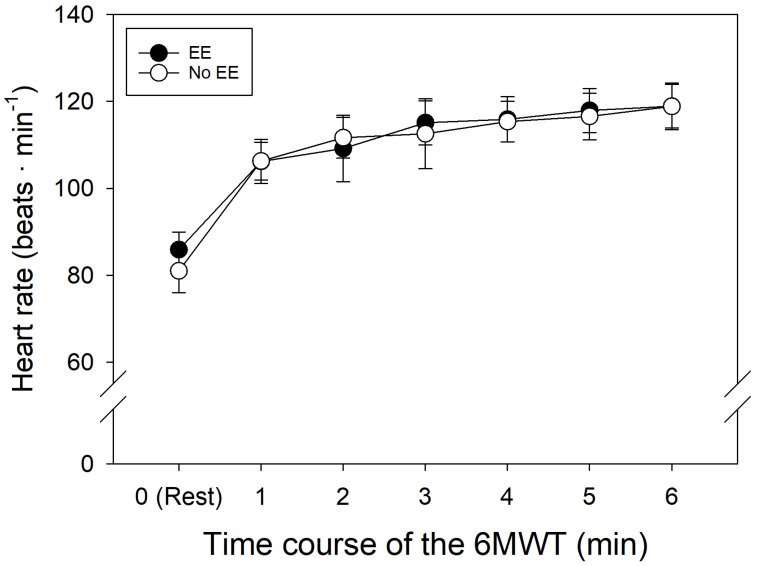
Heart rate over time along 6MWT in patients with and without EE.

Despite similar basal HR and final HR during the 6MWT in both groups (*p* = 0.228; Figure 2), the increased levels of [Hb] and Hct in subjects with EE do not alter their response to submaximal physical activity, allowing for adequate daily activities in a hypoxic environment [26]. However, oxygen saturation was slightly lower in the subjects with EE (Figure 1). This could be due to the reduced capillary transit time in lung vessels from the pulmonary hypertension associated with EE [18,30]. It is also important to mention that the excessive increase in [Hb] and Hct levels in many cases is not associated with the presence of symptoms [22].

Performance of 6MWT for residents of La Rinconada showed minimal deviation from the reference parameters at sea level (~600 m) [31]. The sensation of dyspnea and fatigue was similar in both control and EE groups and the Borg scale showed that subjects with EE may have a lower sensation of dyspnea than subjects without EE (Table 4). Although these parameters never reached statistical significance, a higher sample size may provide further insight into the differences observed for dyspnea and fatigue perception.

While no statistically significant differences were observed between the two groups, the dyspnea score evaluated through the Borg scale tended to be lower in subjects with EE relative to the control group (Table 4) (*p* = 0.27). Fatigue was also similarly reported in both groups (*p* = 0.33), suggesting that subjects with EE have a similar effort perception to subjects without EE during the 6MWT.

**Figure 3:**
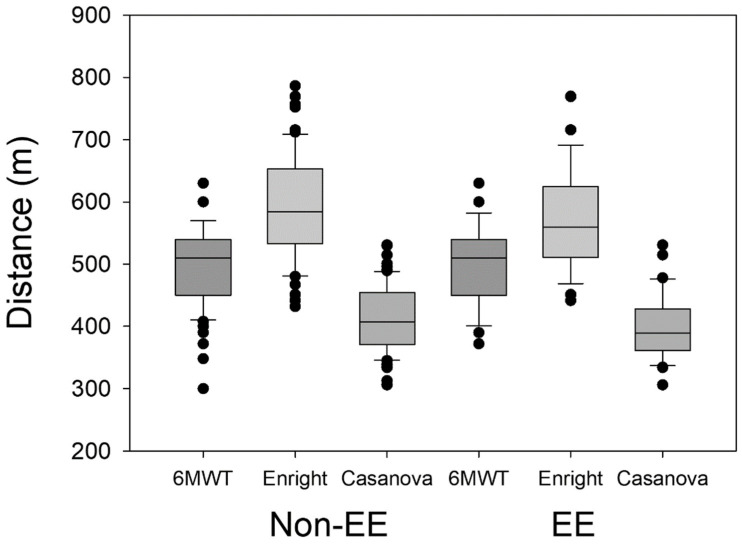
Actual traveled distance and calculated distances.

**Figure 4:**
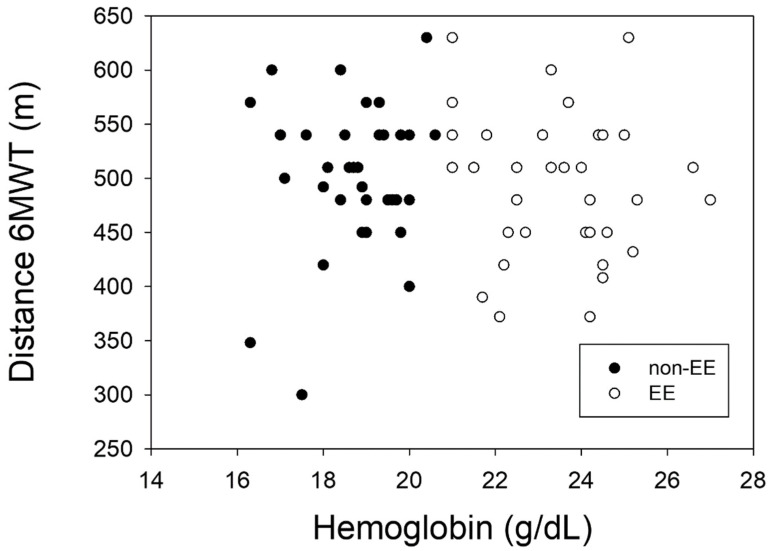
Relationship between actual traveled distance and Hb values.

Additionally, the Enright and Casanova equations have been used to compare the theoretical distance traveled with the values obtained in the present study. The Enright formula [12] overestimated the values obtained in the present work, while the Casanova formula underestimated these values [10]. In the present study, we show that the formulas used do not allow us to accurately predict the distance traveled by the residents of La Rinconada (Figure 3 and Figure 4), and like other studies it can be observed that the hypoxic environment is not a limitation for the performance of submaximal physical activity in subjects adapted to conditions of severe hypoxia [27].

The basic output of the 6MWT showing similarities between subjects with EE compared to no EE reinforces the importance to study other physiological changes that may allow high-altitude inhabitants to carry out daily activities. This includes studying negative physiologic changes that do not support physical effort in hypoxia. In addition, functional studies to better characterize the hemodynamic response during physical activity is also required to better characterize the maximum physical capacity of the subjects, allowing for a better comparison to subjects who live in areas of lower altitude. An important characteristic of residents in hypoxic environments is pulmonary hypertension [6,18]. Some studies have considered the 6MWT as the main endpoint in clinical trials for pulmonary hypertension [32]. In this case, it would be important to consider this method for the evaluation of patients in high-altitude regions due to the high prevalence of this pathology.

The overall findings in this study allow us to understand how the human body can adapt to severe environmental conditions. In high-altitude regions, low barometric pressure limits the uptake of oxygen and decreases the alveolar–arterial oxygen gradient. However, compensatory mechanisms are activated to favor oxygen uptake at the cellular level, allowing for an adequate hemodynamic response during physical activity [6]. The La Rinconada population, like other high-altitude populations including the Sherpa people, have a great capacity for physical effort, which may be related to their genetic make-up, that allows them to have a higher tolerance of the environment. It is known that hypoxia regulates oxygen-sensitive genes of cells. The hypoxic impact on genes facilitates the production of energy as well as compensatory mechanisms in order to avoid oxidative damage. This improves muscle energy bioavailability and regulates lactic acid production. Some of these genes have already been identified, including the PPARA gene [33]. Although the 6MWT test is generally used for the follow-up and prognosis of cardio-respiratory diseases, this test allowed us to evaluate the potential impact of excessive increases in [Hb] and Hct on the physical capacity of high-altitude residents. We conclude that subjects with EE do not present with marked limitations in carrying out their daily activities compared to subjects with normal [Hb] and Hct values. Our findings agree with previous studies showing a slight decrease in physical capacity in subjects with Chronic Mountain Sickness at 4300 m [34].

## 5. Conclusions

The reference values used at sea level in the 6MWT can be valid and indicative for the evaluation of individuals who live permanently at high altitudes irrespective of their Hct and [Hb] values. EE does not significantly alter the results of the 6MWT, showing no major hemodynamic changes in subjects who live permanently in a severe hypoxic environment.

## Figures and Tables

**Table 1:** Demographic, anthropometric, and hematological characteristics.

	EE	Control	*p*-Value	Cohen’s d
N (%)	35 (49.3%)	36 (50.7%)		
Age (years)	47 ± 11.0	43 ± 12.0	0.195	−0.30
BMI (kg·m^−2^)	26.79 ± 3.34	26.71 ± 3.45	0.662	−0.02
Body weight (kg)	73.2 ± 10.5	72.5 ± 9.0	0.765	0.07
Height (cm)	165.7 ± 5.8	164.6 ± 5.7	0.390	0.18
[Hb] (g·dL^−1^)	23.4 ± 1.6	18.7 ± 1.2	**<0.001**	−1,70
Hct (%)	73.6 ± 5.9	60.4 ± 7.1	**<0.001**	−1.42

Data mean ± SD. EE, excessive erythrocytosis; BMI, body mass index; [Hb], hemoglobin concentration; Hct, hematocrit. Bold font indicates a *p*-value statistically significant (*p* < 0.05).

**Table 2:** Cardiovascular parameters before (basal) and at the end of the 6 min walking test.

		Basal	End	*p*-Value	Δ *	*p* * Value
SBP (mmHg)	EE	128 ± 14	138 ± 16	0.280	9 ± 12	0.052
	Control	118 ± 12	134 ± 15		15 ± 14	
DBP (mmHg)	EE	84 ± 11	89 ± 14	0.385	5 ± 13	0.395
	Control	79 ± 8	87 ± 9		8 ±10	
HR (beats·min^−1^)	EE	85 ± 12	119 ± 15	0.872	33 ± 12	0.228
	Control	82 ± 15	119 ± 16		37 ± 14	
SpO_2_ (%)	EE	80 ± 5	74 ± 5	**0.001**	6 ± 4	0.257
	Control	83 ± 3	78 ± 5		5 ± 4	

Data are mean ± SD. HR, Heart rate; SBP, systolic blood pressure; DBP, diastolic blood pressure; SpO_2_, peripheral arterial oxygen saturation; Δ*, exercise vs. basal difference, *p* * for the differences between EE and control groups. Bold font indicates a *p*-value statistically significant (*p* < 0.05).

**Table 3:** Arterial oxygen saturation during the 6MWT.

		Rest	Min 1	Min 2	Min 3	Min 4	Min 5	Min 6
SpO_2_ (%)	EE	80 ± 5	77 ± 4	74 ± 4	73 ± 11	74 ± 4	74 ± 5	73 ± 7
	Control	82 ± 3	81 ± 4	79 ± 5	79 ± 4	78 ± 5	78 ± 4	77 ± 7
HR (beats·min^−1^)	EE	86 ± 12	106 ± 15	109 ± 23	115 ± 15	116 ± 15	118 ± 15	119 ± 15
	Control	83 ± 16	108 ± 13	112 ± 14	113 ± 24	116 ± 14	117 ± 16	119 ± 16

Data are mean ± SD.

**Table 4:** Distance traveled in 6MWT and Borg scale values.

			Percent Difference (%)	*p*-Value	Cohen’s d
Distance (m)	EE	494 ± 66	1.58%	0.433	0.13
	Control	503 ± 68			
Borg (dyspnea)	EE	3 ± 2	8.86%	0.275	0.19
	Control	4 ± 2			
Borg (fatigue)	EE	5 ± 2	7.76%	0.331	0.21
	Control	5 ± 3			

Data are mean ± SD.

## Data Availability

The raw data supporting the conclusions of this article will be made available by the corresponding authors, upon reasonable request.
